# Supplementary material for: The effect of supragingival glycine air polishing on periodontitis during maintenance therapy: a randomized controlled trial
Source: PeerJ. 2018 Feb 12;6:e4371. doi: 10.7717/peerj.4371 (PMC5813589; doi:10.7717/peerj.4371)
Supplement: Supplemental Information 2 — SGAP group, supragingival glycine air polishing; SUSP, supragingival ultrasonic scaling and polishing. [file peerj-06-4371-s002.docx]

**Protocol**

**1 Participants**

23 patients treated for chronic periodontitis and involved in an supportive periodontal therapy (SPT) program at the Department of Periodontology, Peking University School and Hospital of Stomatology, China, were invited for the study that was conducted between August 2011 and March 2013. The patients included 8 males and 15 females, with their age ranging from 28 to 72 years old.

Inclusion criteria were as followed:

- Patients had received completed periodontal treatment(including that had been treated by periodontal surgery more than six months before) with periodontal probing depth less than 5mm.
- Remaining more than 20 teeth
- Without systemic disease.
- No smoking.

The following criteria were used to exclude subjects from participating:

- Pregnancy or lactation.
- Scaling and root planning within 3 months preceding the start of the trial.
- Antibiotic therapy within 3 months preceding the start of the trial.

**2 Interventions**

According to split-mouth design ,one side of a mouth was randomly assigned to the experimental group with glycine powder air-polishing therapy while the other side was the control group with ultrasonic scaling and polishing paste therapy. Random number table was used to allocate the experimental group and the control group.

Supragingival air polishing was applied in the experimental group with

the use of 65 μm amino acid glycine powder(airfloe polishing soft, EMS, Switzerland), AIR-FLOW handy2(EMS,Switzerland) and Air-Flow Masters (EMS,Switzerland).Ultrasonic scaling was applied in the control group with the use of ultrasonic instrument(Satelec, French) , contra-angle handpiece(NSK,Japan)and polishing paste (Ivoclar, Fürstentum Liechtenstein).

Fig 1. Flow-chart diagram of the study.


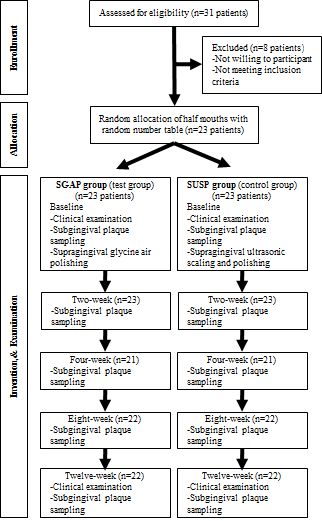


**2 Clinical examination**

Clinical periodontal examination was performed at baseline before treatment and the endpoint at 12 weeks. It was carried out by an experienced periodontist, whose self-consistency test showed substantial consistency, with 96% of the sites differing 1 mm or less for pocket depth measurements. The examiner was masked with the general information and treatment information of recruited subjects. Criteria for periodontal clinical examination were as follows:

1. Plaque index(PLI): Silness and Löe’s([1](#_ENREF_1))
2. Probing depth(PD): The distance from the bottom of periodontal pockets to gingival margin using Williams probe.
3. Bleeding index(BI): Mazza’s([2](#_ENREF_2))
4. Bleeding on probing(BOP): The sites with BI≥2 were recorded as BOP-positive.

Participants received oral hygiene instruction(OHI) after clinical examination every time.

**3 Sample collection**

1. Sampling of the subgingival microbiota at each investigational site was performed before treatment and at 2 weeks,4 weeks,8 weeks and 12 weeks after the treatment. The mesiobuccal sites of 14,11,21,24,36,46 were sampled. The flow chart is showed in figure 1.
2. Isolate the sampled area with cotton rolls,and then dry it with gentle air .
3. Clean the supragingival area with cotton pellets.
4. Collect subgingival dental plaque with sterile Gracey curettes in the mesiobuccal sites of two upper first incisor,two upper first premolar,two lower first molar,and transfer the samples into sterile Eppendorf tubes,respectively.
5. Seal the tubes with rubber seal strip.
6. 5. Store the samples in -80℃ biological sample preservation refrigerator.

**4 DNA extraction**

1. Take out the samples from -80℃ biological sample preservation refrigerator.
2. Add 500 μl TE buffer(10 mM Tris-HCL, 1 mM EDTA,pH 7.6 ) to the tube,and vortex it until the plaque was broken up.Centrifuge at 13000rpm（r=5.5cm) for 5 min. Transfer top layer to a new tube and remain precipitate.
3. Wash twice again,and remain precipitation - 20 ℃ cryopreserved.
4. Add 180 μl lysozyme to the tube,and water bath at 37℃ overnight.
5. Isolate genomic DNA using microscale genomic DNA extraction kit(TIANGEN,China) , according to the manufacturer’s instructions.
6. Check concentration and purity of DNA by spectrophotometric analysis.

**5 Polymerase chain reaction (PCR) measurements**

The samples were analyzed for the detection of Porphyromonas gingivalis (Pg),Tannerella forsythia(Tf),Treponema denticola(Tf),Campylobacter rectus(Cr), Prevotella intermedia(Pi), Prevotella nigrescens (Pn), Fusobacterium nucleatum (Fn), Parvimonas micra (P.micra) using PCR and agarose gel electrophoresis. The definitive primers is displayed in table 1,and the reaction mixtures and Thermocycling condition are showed in table 2, table 3 and table 4, respectively[1-3]. The PCR cycling was performed by the GeneAmp PCR system 2700 (ABI, South San Francisco, CA, USA).

*Table 1*, Definitive primers for PCR

|  |  | Primer sequence(5'-3') | Base position (length) | References |
| --- | --- | --- | --- | --- |
| *Porphyromonas gingivalis* | | | | |
|  | Forward | AGG CAG CTT GCC ATA CTG CG | 729-1,132 (404) | Ashimoto *et al.* |
|  | Reverse | ACT GTT AGC AAC TAC CGA TGT |  |  |
| *Tannerella forsythia* | | | | |
|  | Forward | GCG TAT GTA ACC TGC CCG CA | 120-760 (641) | Ashimoto *et al.* |
|  | Reverse | TGC TTC AGT GTC AGT TAT ACC T |  |  |
| *Treponema denticola* | | | | |
|  | Forward | TAA TAC CGA ATG TGC TCA TTT ACA T | 193-508 (316) | Ashimoto *et al.* |
|  | Reverse | TCA AAG AAG CAT TCC CTC TTC TTC TTA |  |  |
| *Fusobacterium nucleatum* | | | | |
|  | Forward | AGGGCATCCTAGAATTATG | 190–1,006(817) | Baumgartner *et al.* |
|  | Reverse | GGGACACTGAAACATCTCTGTCTCA |  |  |

*Table 2*,Pg，Tf，Td reaction mixture.

| Reagent | volume (μl) |
| --- | --- |
| Template | 2 |
| 10×buffer （Mg plus） | 2.5 |
| d NTP(2.5mM) | 2 |
| Taq DNA polymerase(5U/ul) | 0.2 |
| Primer(10µM) | 1 |
| Dd H2O | 17.3 |
| Total | 25 |

Thermocycling for Pg，Tf，Td，Cr

95℃ 2 min;

95℃ 30 sec, 60℃ 1 min, 72℃ 1 min, 36 cycles

72℃ 2 min

*Table 3*, Fn reaction mixture.

| Reagent | volume (μl) |
| --- | --- |
| Template | 2 |
| 10×buffer (Mg plus) | 2.5 |
| d NTP(2.5mM) | 2 |
| Taq DNA polymerase(5U/ul) | 0.2 |
| Primer(10 µM) | 1 |
| Dd H2O | 17.3 |
| Total | 25 |

Thermocycling for Fn

94℃ 1 min

94℃ 1 min, 60℃ 1 min, 72℃ 90 sec, 35 cycles

72℃ 10 min

### 6 Agarose gel electrophoresis

1. Measure 30 mL of 1X TAE(Solarbio,Beijing,China) buffer in a graduated cylinder and pour it into a 100 mL flask.

2. Weigh 0.45 grams of electrophoresis grade agarose(Solarbio, Beijing, China) and add it to the buffer solution in the flask and swirl it gently.

3. No keeping the cap of flask too tight .

4.Adjust the level of fire to mediate-high and place the flask into a microwave,and turn off the door.

5.Heat 1.5 min, take out the flask by glove.

6.Cool the agarose to about 50-55°

7.Add 1.5 μl goldview (Solarbio,Beijing,China) to the agarose and swirl it.

8.Place the gel tray into the gel box so that the ends of the tray are blocked by the sides of the box perpendicular to the ends of the box. Place the comb in the slots on one end of the tray.

9.Pour the cooled agarose into the tray and there is an equal amount of buffer solution in either chamber .The thickness is about 4mm. Let it cool until it is solid about 30minutes.

10.Carefully pull upward on the comb to remove it once the agarose has solidified.Remove the tray from the box into the electrophoresis box.and so there is about 2-3 mm of solution above the gel.

11.Mix 5μl PCR product with 1μl loading buffer(Solarbio,Beijing,China) ,

Introduce the mixture solution and standard DNA DL2000(TaKaRa Biotechnology, Dalian, PR China) into the bottom of the well.Set voltage as 130V,time as 30 minutes.

12.Photograph the gel under 300 nm ultraviolet illumination.

**References:**

[1]. Ashimoto, A., et al., Polymerase chain reaction detection of 8 putative periodontal pathogens in subgingival plaque of gingivitis and advanced periodontitis lesions. Oral Microbiol Immunol, 1996. 11(4): p. 266-73.

[2]. Baumgartner, J.C., et al., Geographical differences in bacteria detected in endodontic infections using polymerase chain reaction. J Endod, 2004. 30(3): p. 141-4.

[3]. Cortelli, J.R., et al., Detection of periodontal pathogens in newborns and children with mixed dentition. Eur J Clin Microbiol Infect Dis, 2012. 31(6): p. 1041-50.

[4]. Morillo, J.M., et al., Quantitative real-time PCR based on single copy gene sequence for detection of Actinobacillus actinomycetemcomitans and Porphyromonas gingivalis. J Periodontal Res, 2003. 38(5): p. 518-24.

1. Silness J, Loe H. PERIODONTAL DISEASE IN PREGNANCY. II. CORRELATION BETWEEN ORAL HYGIENE AND PERIODONTAL CONDTION. Acta Odontol Scand. 1964;22:121-35.

2. Mazza JE, Newman MG, Sims TN. Clinical and antimicrobial effect of stannous fluoride on periodontitis. J Clin Periodontol. 1981;8(3):203-12.
